# Multi-transmitter characteristics and functional specialization of oxytocin neuron subpopulations in zebrafish

**DOI:** 10.1242/jeb.252228

**Published:** 2026-05-13

**Authors:** Sian-Tai Liu, Bo-Kai Liao, Meng-Shin Shaio, Yen-Lin Chen, Yu-Fan Huang, Ming-Yi Chou

**Affiliations:** ^1^ Department of Life Science, National Taiwan University, Taipei 10617, Taiwan; ^2^ Department of Aquaculture, National Taiwan Ocean, University, Keelung 202301, Taiwan; ^3^ Research Laboratory Section, Offices of Health Science Research, Faculty of Medicine Ramathibodi Hospital, Mahidol University, Bangkok 10400, Thailand

**Keywords:** Isotocin/oxytocin, Vasotocin, Arginine vasopressin, Nonapeptides, *Danio rerio*

## Abstract

Teleost oxytocin (OXT) neurons are much fewer in number than those reported in mammals, suggesting that individual neurons may support a broader range of functions. This phenomenon raises the possibility that co-expression of multiple neurotransmitters allows teleost OXT neurons to support various functions. To test this possibility, we systematically examined neurotransmitter characteristics and activity patterns of OXT neuron subtypes in adult zebrafish brain. We found that OXT neurons are distributed across the parvocellular preoptic nucleus (anterior, PPa; posterior, PPp) and the periventricular posterior tuberculum (TPp), segregating by soma size into parvocellular (≤11.62 μm) and magnocellular (>11.62 μm) subtypes. The number of the parvocellular OXT neurons follows an anterior-to-posterior enrichment gradient, whereas magnocellular neurons display an inverse posterior-to-anterior gradient. The OXT neurons co-express glutamatergic, GABAergic and cholinergic neuron markers and show partial co-localization with vasotocin (AVT), while lacking dopaminergic and serotonergic synthesis markers. We also quantified activity-dependent p-S6 expression during mating. p-S6 increased in parvocellular OXT neurons of the PPa and PPp, but not in magnocellular neurons of these regions or in either neuronal type in the TPp. Together, these findings suggest that zebrafish OXT neurons may coordinate behavioral and endocrine functions through multi-transmitter expression and partial AVT co-expression. These results further indicate that anatomically distinct OXT neuron subtypes differ in their functional recruitment, with parvocellular neurons in the PPa and PPp possibly involved in mating-related interactions.

## Teaser

**Summary:** Zebrafish oxytocin neuron subpopulations exhibit distinct multi-transmitter identities and functional specializations during reproductive social behavior.

## INTRODUCTION

In mammals, oxytocin (OXT) neurons influence vertebrate physiology through a dual-function pathway of signaling by releasing OXT both as hormone and as synaptic neurotransmitter in the central nervous system. There are approximately 8000 OXT neurons in the rat hypothalamus and up to 50,000 in humans. These neurons are differentiated into functionally specialized magnocellular populations located in the paraventricular nucleus (PVN) and supraoptic nucleus (SON), along with smaller parvocellular OXT populations in the PVN that project broadly to limbic and brainstem targets (Chini et al., 2017). This cellular diversification and division of labor give rise to the high degree of neurofunctional complexity characteristic of the mammalian OXT system.

As a hormone, OXT is synthesized in the magnocellular neurons of the hypothalamus and released from the posterior pituitary into systemic circulation. It then binds to peripheral G-protein coupled receptors to mediate uterine contractions during parturition and milk ejection during lactation (Gimpl and Fahrenholz, 2001). As neurotransmitter, OXT is synthesized by hypothalamic parvocellular neurons, which send projections to diverse brain regions (Althammer and Grinevich, 2017; Ludwig and Pittman, 2003) and act as a neuromodulator (Raggenbass, 2001). Additionally, it influences the social and emotional behaviors in part by modulating hippocampal circuitry (Donaldson and Young, 2008; Heinrichs et al., 2009; Meyer-Lindenberg et al., 2011). In the hippocampal CA1 region, OXT enhances GABAergic transmission by depolarizing a specific subclass of interneurons, resulting in increased inhibitory input to pyramidal neurons and reduced network excitability (Maniezzi et al., 2019; Zaninetti and Raggenbass, 2000). By fine-tuning the excitation–inhibition balance in the hippocampal networks, such modulation contributes to the regulation of social cognition and anxiety-related behaviors (Donaldson and Young, 2008; Heinrichs et al., 2009). OXT also increases pain thresholds and participates in osmoregulation (Mekhael et al., 2023; Quintana and Guastella, 2020).

By contrast, teleosts represent a more basal vertebrate lineage, and species such as zebrafish (*Danio rerio*) possess far fewer OXT neurons, with only ∼300 cells identified in the preoptic area (POA) (Chuang et al., 2021). In teleosts, the oxytocin homologue, previously referred to as isotocin, and the arginine vasopressin (AVP) homologue, vasotocin (here referred to as AVT), form the conserved nonapeptide pair analogous to mammalian OXT and AVP (Theofanopoulou et al., 2021). This stark numerical and anatomical simplicity has led to the hypothesis that roles later partitioned among distinct mammalian OXT subpopulations were executed by a small, multifunctional OXT population in teleosts, requiring each neuron to support a broader range of physiological and behavioral functions as part of an ancestral organizational strategy.

Teleost OXT signaling is responsible for conspecific recognition and social behaviors (Landin et al., 2020; Nunes et al., 2020, 2021), whereas hypothalamic OXT projections to brainstem circuits mediate defensive escape responses to noxious stimuli (Wee et al., 2019). In medaka, OXT governs sexually dimorphic mate choices and male preference in females in mating behaviors (Yokoi et al., 2019). OXT is also found to regulate cellular reproductive processes including gametogenesis and spawning through hypothalamic–pituitary–gonadal coordination, and controls osmoregulation via hypothalamic–pituitary modulation of branchial ionocyte function (Chou et al., 2010; Chuang et al., 2021; Mennigen et al., 2022; Tong et al., 2025; Zanardini and Habibi, 2025). These findings suggest that the extensive functional versatility of teleost OXT neurons, despite their small population of only ∼300 cells, is supported not only by subpopulation differentiation in a single neurotransmitter system, but also by their multi-transmitter characteristics and functional specialization, which together enable them to regulate a wide range of physiological and behavioral processes.

Building on this idea, recent work demonstrated that OXT and corticotropin-releasing factor (CRF) peptidergic neurons in zebrafish co-release glutamate (92.7% and 95.6% *vglut2a*-positive cells, respectively), whereas somatostatin (SST) neurons show minimal glutamatergic identity (11.8% *vglut2a*-positive cells) (Lovett-Barron et al., 2020). This finding suggests that distinct neurotransmitter co-expression patterns in peptidergic neurons may be associated with different behavioral functions. In addition, zebrafish OXT neurons also co-express other neuropeptides within the neurosecretory POA, further increasing their molecular and functional diversity (Herget and Ryu, 2015; Wircer et al., 2017). However, most of these co-expression analyses were performed in embryos or larvae, which lack the full repertoire of adult social and motivational behaviors. Even so, developmental perturbation or ablation of a discrete population of posterior tuberculum OXT neurons co-expressing CRH has been shown to produce long-term deficits in adult social preference (Wircer et al., 2017). Nevertheless, it is still unclear how anatomically defined OXT neuron subtypes in the adult brain use multiple neurotransmitters or neuropeptides to regulate ongoing social and motivational behaviors.

In the present study, we investigated the topology and distribution of magnocellular and parvocellular OXT neurons in the adult zebrafish brain. We demonstrated that zebrafish OXT neurons express markers of glutamatergic, GABAergic and cholinergic neurons, and that some OXT neurons also express AVT. We further demonstrated that only a subset of parvocellular OXT neurons, rather than all OXT neurons, is responsible for mating behaviors. Our results support the notion that zebrafish OXT neurons are heterogeneous, form distinct subpopulations and express different types of neuronal markers, which enables them to regulate multiple behaviors.

In mammals, OXT and AVP neurons in the PVN and SON are generally regarded as largely distinct but partly overlapping populations, and clear OXT–AVP double-labelled cells are only rarely observed (Soumier et al., 2021). However, whether a comparable degree of segregation is present in teleosts remains unclear. In the present study, we performed a comparison in the mouse hypothalamus to visualize how the OXT–AVP relationship in zebrafish compares with that in the mammalian brain.

## MATERIALS AND METHODS

### Animals

Adult zebrafish [*Danio rerio* (Hamilton 1822)] of the Tg(*oxt*:EGFP) transgenic line, which expresses EGFP under the control of a 644 bp *oxt* promoter fragment (Gutnick et al., 2011; ZFIN: ZDB-TGCONSTRCT-111103-1), were used in all experiments. This line has been employed to study OXT neuron structure and function in adult zebrafish (Chuang et al., 2021; Gutnick et al., 2011). Adult zebrafish aged 10–12 months were sourced from controlled laboratory breeding at the Department of Life Science, National Taiwan University. The experimental population consisted of approximately equal proportions of males and females (male:female ratio ≈1:1), with individuals ranging from 2.8 to 3.3 cm in total body length. Fish were maintained in 10 l holding tanks at a density of 40 fish per tank under a standardized photoperiod of 14 h:10 h light:dark. Tank water was prepared by dechlorinating municipal tap water using a 3M™ S301 Water Filtration System (3M™, St Paul, MN, USA). Environmental conditions were maintained at a constant temperature of 28±0.5°C with a light intensity of approximately 150 lux. Water chemistry was kept at neutral pH (7.0) and monitored daily, with regular water exchanges performed to maintain optimal quality and dissolved oxygen levels. All animal care and experimental procedures were conducted in accordance with institutional animal welfare guidelines and received ethical approval from the Institutional Animal Care and Use Committee of National Taiwan University (approval number: NTU-113-EL-00110).

Adult C57BL/6J wild-type mice were obtained from the National Laboratory Animal Center (NLAC, Taipei, Taiwan). Mice were housed under a 12 h:12 h light:dark cycle (lights on 08:00–20:00 h) at 22±1°C with *ad libitum* access to food and water. Mouse procedures were approved by the Institutional Animal Ethics Committee of National Taiwan University (NTU-112-EL-00128) and followed NLAC guidelines. Mouse brain tissue was used solely for qualitative anatomical comparison of OXT and AVP neuron distributions and was not included in quantitative analyses.

To prepare brain tissue for neuroanatomical mapping, neurotransmitter and AVT co-expression analysis, and p-S6 activity mapping (see Results), adult Tg(*oxt*:EGFP) zebrafish were anesthetized with MS-222 (150 mg l^−1^) and euthanized by rapid cervical transection. Brains were rapidly dissected in ice-cold PBS (pH 7.4), fixed in 4% paraformaldehyde (PFA) in PBS, rinsed thoroughly and embedded in 2% agarose. Serial coronal sections [80 µm for fluorescence *in situ* hybridization (FISH), 100 µm for immunohistochemistry (IHC)] were cut on a vibrating microtome and stored in PBS until further processing. Because relatively thick free-floating sections were used to preserve the three-dimensional morphology of OXT neurons and their processes, all fluorescent images were acquired using laser-scanning confocal microscopy with optical sectioning to minimize out-of-focus fluorescence. Detailed embedding and vibratome settings are provided in Supplementary Materials and Methods.

For mouse brain sections, coronal slices (80 µm) encompassing the SON and PVN were processed for OXT, AVP and tyrosine hydroxylase (TH) IHC using standard blocking, primary and secondary antibody incubations, and DAPI counterstaining. Detailed antibody information and staining conditions are provided in Supplementary Materials and Methods.

All fluorescently labeled sections were imaged on a laser-scanning confocal microscope (Leica SP5, Leica Microsystems) using an inverted microscope frame (Leica DMI6000 CS). *Z*-stacks were acquired with identical acquisition parameters within each experiment, and orthogonal views (*x*–*z*, *y*–*z*) were inspected to confirm signal co-localization. Image analysis, including cell counting and soma measurements, was performed in LAS X software (Leica Microsystems) as described below.

### Neuroanatomical mapping of OXT neuron subtypes

To characterize the neuroanatomical organization of magnocellular and parvocellular OXT neurons, we mapped OXT-GFP^+^ somata across the parvocellular preoptic nucleus, anterior (PPa) and posterior (PPp), and the periventricular posterior tuberculum (TPp). For each animal, serial coronal sections spanning the rostro-caudal extent of these regions were imaged with confocal microscopy, and Nissl counterstaining was used to delineate anatomical boundaries.

OXT-GFP^+^ cells were identified and segmented in maximum-intensity projections, and soma diameter was measured in LAS X software (Leica Microsystems). Cells were classified as parvocellular or magnocellular based on an empirically determined size threshold derived from the bimodal distribution of soma diameters (11.62 µm).

For quantitative analysis of OXT neuron number and soma size, cell counting was performed using a region-of-interest (ROI) approach. In the PPa and PPp, sections were divided into dorsal and ventral compartments based on the midline of the OXT neuron immunofluorescent signal. Within each compartment, multiple 45×45 µm ROIs were randomly placed in areas containing OXT neurons, and only ROIs that contained at least one OXT neuron were included in the analysis. Across animals, the number of sampled ROIs corresponded to approximately 40% of the total OXT neuron-containing area in each compartment. All OXT-GFP^+^ cells within the selected ROIs were counted and their soma diameters were measured. In the TPp, the total OXT neuron population was small (*n*<20 per brain), so all OXT-GFP^+^ cells in this region were counted and their soma diameters were measured rather than sampled by ROIs.

### Neurotransmitter and AVT co-expression analysis

To determine the neurotransmitter identity and AVT co-expression profiles of OXT neurons, we combined FISH for glutamatergic, GABAergic, cholinergic and AVT markers with anti-GFP IHC in adult Tg(*oxt*:EGFP) brains. Total RNA was extracted from adult zebrafish brains and reverse-transcribed to cDNA, and DIG-labeled antisense riboprobes were synthesized against *gad1b*/*gad2* (GABAergic), *slc17a6a*/*slc17a6b* (glutamatergic), *chata*/*chatb* (cholinergic) and *avt*. The targeted nucleotide ranges and full probe synthesis protocol are provided in Supplementary Materials and Methods.

Fixed vibratome sections were processed with a standard hybridization protocol, including graded methanol dehydration/rehydration, protease treatment, hybridization in formamide-based buffer, high-stringency SSC washes, and peroxidase-based tyramide signal amplification (TSA). Detailed buffer compositions and wash schedules are described in Supplementary Materials and Methods. After FISH, sections were blocked and incubated with anti-GFP primary antibody followed by fluorophore-conjugated secondary antibodies to visualize OXT-GFP^+^ neurons. Nuclei and cytoarchitecture were counterstained with a fluorescent Nissl dye.

Confocal *z*-stacks were acquired from the PPa, PPp and TPp, and OXT-GFP^+^ cells were assessed for co-localization with each FISH signal in 3D using orthogonal views to avoid false-positive overlap. In each brain, we qualitatively evaluated which subsets of OXT neurons expressed the different neurotransmitter markers and *avt*, and described how co-expressing cells were distributed across parvocellular and magnocellular subtypes and across brain regions.

### Mating behavior and p-S6 activity mapping

To assess activity-dependent recruitment of OXT neuron subtypes during mating, we used a standardized zebrafish mating assay combined with p-S6 IHC. Twelve hours before testing, sexually mature male–female pairs were placed into partitioned 1.2 l test tanks filled with system water at 28±0.5°C. A transparent acrylic divider allowed visual but not physical contact. At the onset of the light phase, the divider was removed to permit courtship and spawning; behavior was observed for 30 min.

Pairs were included in the mating-behavior group only when they met predefined criteria, namely that males exhibited at least three pursuit episodes lasting ≥10 s and females displayed at least one egg-release (spawning) event. Pairs that did not meet these criteria were excluded from the mating-behavior group, and fish were anesthetized 50 min after the onset of mating behavior so that brains could be collected and fixed for IHC at a time point expected to capture peak p-S6 phosphorylation. For the non-mating condition, we used sexually mature males and females taken directly from the high-density home tank (approximately 40 fish in 10 l). These fish were sampled at the same time of day as the mating group.

For p-S6 analysis, brains were fixed in 4% PFA for 2 h at room temperature, cryoprotected in 4% agarose, embedded and cut into 100-µm coronal sections. Sections spanning the PPa, PPp and TPp were assessed for immunofluorescence with anti-p-S6 and anti-GFP antibodies, and OXT-positive cell bodies were counted in confocal *z*-stacks to determine the proportion that was p-S6-positive. Detailed antibody information, blocking conditions and wash protocols are provided in Supplementary Materials and Methods.

Confocal images spanning the PPa, PPp and TPp were acquired with identical settings across groups. For each brain, OXT-GFP^+^ cells were classified as p-S6^+^ or p-S6^−^, and the proportion of activated cells was quantified separately for parvocellular and magnocellular OXT neurons in each region. Counts were performed bilaterally across matched sections and averaged per animal.

### Statistical analysis

All data are presented as means±s.e.m. The normality of data distribution within each group was assessed using the Shapiro–Wilk test prior to statistical analysis. For comparisons between two independent groups, a two-tailed unpaired Student's *t*-test was applied when data followed a normal distribution; if the normality assumption was violated, the non-parametric Mann–Whitney *U*-test was used instead. Data distributions and variances were inspected to confirm that the assumptions of the parametric tests were reasonably met. All statistical analyses were conducted using GraphPad Prism version 10 (GraphPad Software, San Diego, CA, USA), and a *P*-value <0.05 was considered statistically significant.

## RESULTS

### Anatomical distribution of OXT neurons in adult zebrafish brain

**Fig. 1.**
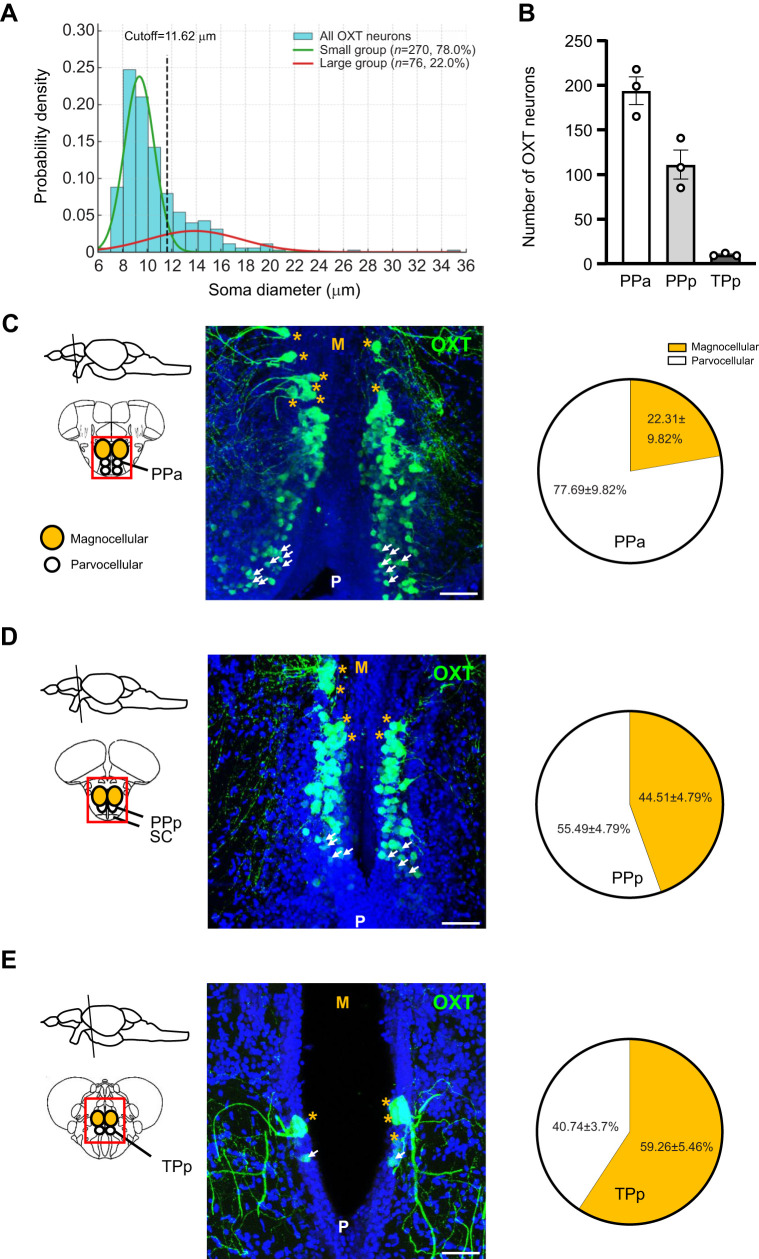
**Distribution of oxytocin (OXT) neurons in adult zebrafish brain.** (A) Gaussian mixture model (GMM) analysis of OXT neuron soma diameter distribution. (B) Total number of OXT neurons in the parvocellular preoptic nucleus, anterior part (PPa), parvocellular preoptic nucleus, posterior part (PPp), and periventricular nucleus of posterior tuberculum (TPp). (C) Representative coronal section through the PPa showing dorsoventral segregation of OXT neuron morphotypes. Left: schematic indicating magnocellular (M, yellow) and parvocellular (P, white) distributions; right: confocal micrograph of OXT neurons (green) with Nissl counterstain (blue). Yellow asterisks denote magnocellular soma; white arrows denote parvocellular neurons. The pie chart shows that magnocellular neurons comprise 22.31±9.82% of the PPa OXT population. (D) Representative coronal section through the PPp showing similar dorsoventral organization as the PPa. Magnocellular neurons (M, yellow asterisks) are distributed dorsally, parvocellular neurons (white arrows) ventrally. The pie chart indicates magnocellular neurons comprise 44.51±4.79% of the PPp OXT population. (E) Representative coronal section through the TPp showing a dispersed distribution of both magnocellular and parvocellular neurons without clear dorsoventral segregation. The pie chart shows magnocellular neurons comprise 59.26±5.46% of the TPp OXT population. All data are presented as means±s.e.m. Scale bars: 50 μm. All quantifications in B–E were obtained from adult fish (*n*=3; one fish per experiment).

OXT neurons were predominantly distributed throughout the POA, particularly in anterior (PPa) and posterior (PPp) parvocellular preoptic nuclei, and sparsely in the periventricular nucleus of the posterior tuberculum (TPp) of the hypothalamus (Fig. 1C–E). OXT neurons were distributed along a rostrocaudal gradient, with the largest population in the PPa (*n*=3 fish, 156.26±45 cells per fish), declining to intermediate levels in the PPp (*n*=3 fish, 88.2±26 cells per fish) and reaching minimal numbers in the TPp (*n*=3 fish, 9.5±1 cells per fish) (Fig. 1B).

To classify OXT neuron subtypes, we measured the soma diameter of all GFP-labeled OXT neurons in the PPa, PPp and TPp and fitted a Gaussian mixture model (GMM) to the resulting size distribution. The GMM revealed two components with an intersection at 11.62 µm, which we used as a threshold to define parvocellular (<11.62 µm) and magnocellular (≥11.62 µm) OXT neurons (Fig. 1A). Previous teleost studies have described a third, gigantocellular class of nonapeptide neurons based on very large soma size. In our zebrafish dataset, OXT neurons of such size were rare and occurred only at the upper end of the large-size distribution. Based on the GMM analysis, these gigantocellular-sized neurons were classified as magnocellular neurons.

In the PPa, small-diameter neurons constituted the predominant population (77.69±9.82%, *n*=3 fish; Fig. 1C). Within the PPa, large-diameter magnocellular neurons (yellow asterisk) were predominantly localized to the dorsal subdivisions, whereas small-diameter parvocellular neurons (white arrow) were preferentially distributed throughout the ventral regions (Fig. 1C). In the PPp, the number of small- and large-diameter neurons showed comparable proportions (small: 55.49±4.79%; large: 44.51±4.79%; *n*=3 fish; Fig. 1D). Magnocellular neurons (yellow asterisk) in the PPp were predominantly localized to dorsal subdivisions, whereas parvocellular neurons (white arrow) were preferentially distributed in ventral regions (Fig. 1D). The TPp region displayed a distinctly different organizational pattern, with magnocellular (large-diameter) neurons constituting 59.26±5.46% of the OXT population (*n*=3 fish; Fig. 1E), demonstrating regional divergence despite the region's limited overall cell number (less than 20 cells per fish). In contrast to the PPa and PPp, the TPp lacked the pronounced dorsoventral segregation of magnocellular and parvocellular OXT neurons. Instead, OXT neurons in the TPp were distributed throughout the nucleus without clear spatial compartmentalization (Fig. 1E).

### Characterization of the neurochemical identity of OXT neurons

**Fig. 2.**
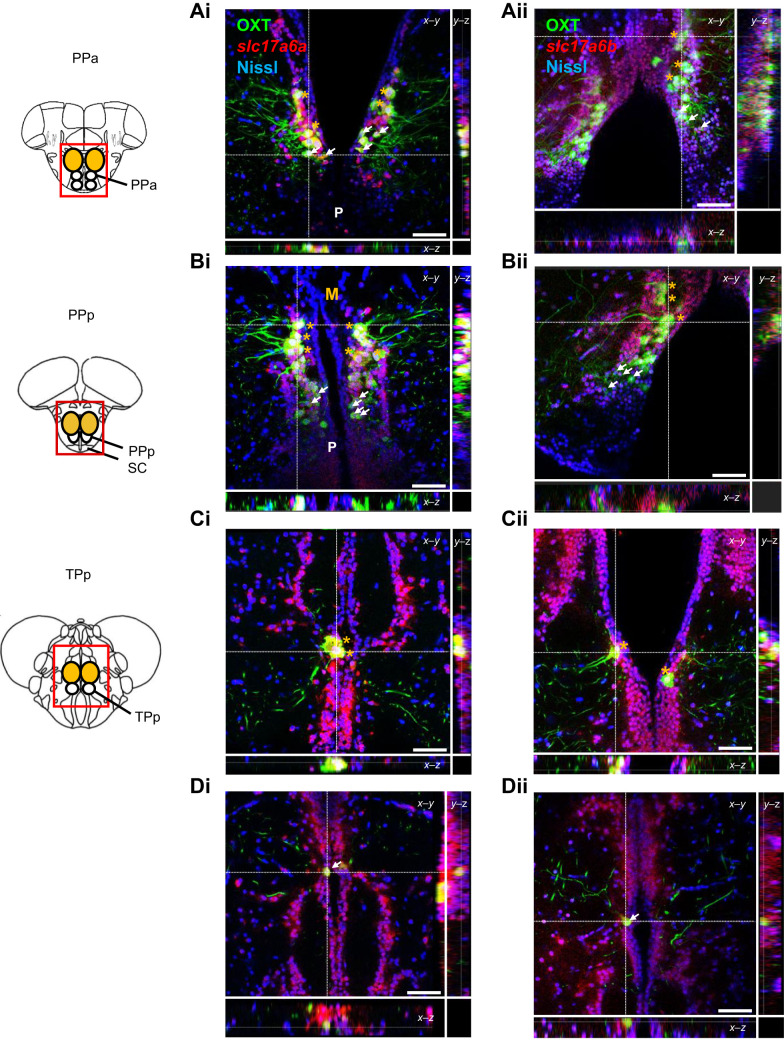
**OXT neurons co-express glutamate across the preoptic and the periventricular nucleus of the posterior tuberculum.** Representative confocal *z*-stack images (with orthogonal projections) of the (Ai,ii) PPa, (Bi,ii) PPp and (Ci,ii,Di,ii) TPp showing OXT neurons (green) co-labeled with glutamatergic markers *slc17a6a* (red; Ai,Bi,Ci,Di) and *slc17a6b* (red; Aii,Bii,Cii,Dii), and neuronal counterstain (Nissl, blue). Yellow asterisks and white arrows denote magnocellular and parvocellular OXT neurons, respectively, that are colocalized with glutamate transporter. M, magnocellular neurons; P, parvocellular neurons; SC, suprachiasmatic nucleus. Scale bars: 50 μm. Images are representative of adult fish (*n*=4; one fish per experiment).

Glutamate is a major excitatory neurotransmitter in the central nervous system. To determine whether OXT neurons exhibit a glutamatergic identity, FISH was utilized to detect the transcripts of vesicular glutamate transporter (*slc17a6a* and *slc17a6b*) within the hypothalamic PPa, PPp and TPp. Confocal images showed that *slc17a6a* signals (red) were colocalized with magnocellular (yellow asterisks) and parvocellular (white arrows) OXT neurons (green) in the PPa (Fig. 2Ai). Orthogonal *x*–*z* and *y*–*z* projections confirmed intracellular overlap. Most OXT neurons observed in the PPa were parvocellular types. In the PPp region, both predominant magnocellular (yellow asterisks) and parvocellular (white arrows) OXT neurons (green) displayed co-expression with *slc17a6a* (red), with signal overlap confirmed in orthogonal projections (Fig. 2Bi). In the TPp, *slc17a6a* was also co-expressed in both magnocellular and parvocellular OXT neurons (Fig. 2Ci,Di), as confirmed by orthogonal *x*–*z* and *y*–*z* projections. A similar pattern of co-expression was observed for *slc17a6b*. Confocal images demonstrated that *slc17a6b* signals (red) consistently overlapped with GFP-labeled OXT neurons (green) in all three hypothalamic regions examined (PPa, PPp and TPp) (Fig. 2Aii–Dii). Orthogonal *x*–*z* and *y*–*z* projections further confirmed that the *slc17a6b* transcripts were present within both parvocellular and magnocellular OXT neurons. As with *slc17a6a*, the number of OXT neurons co-expressing *slc17a6b* in the TPp was lower compared with the PPa and PPp, consistent with the regional distribution observed for OXT neurons. These analyses were performed in adult fish (*slc17a6a*: *n*=4 fish; *slc17a6b*: *n*=4 fish).

**Fig. 3.**
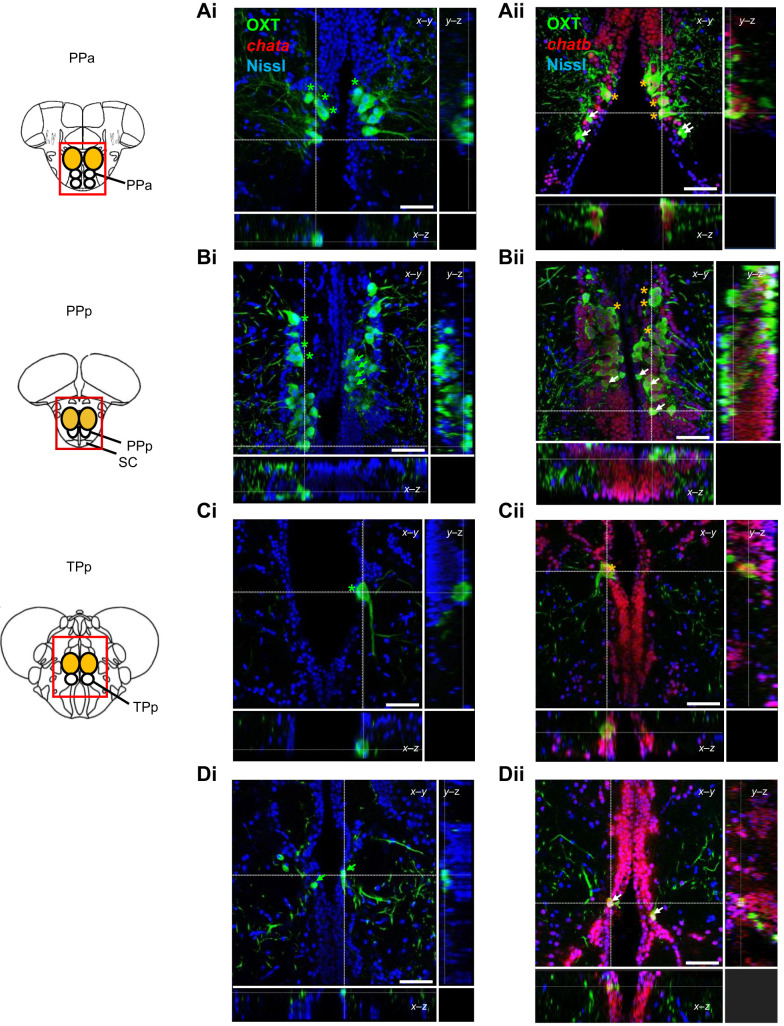
**OXT neurons co-express acetylcholine across the preoptic and the periventricular nucleus of the posterior tuberculum.** Representative confocal *z*-stack images (with orthogonal projections) of the (Ai,ii) PPa, (Bi,ii) PPp and (Ci,ii,Di,ii) TPp showing OXT neurons (green) co-labeled with the cholinergic neurons marker *chatb* (red; Aii,Bii,Cii,Dii) but not *chata* (red; Ai,Bi,Ci,Di), with Nissl counterstain (blue). Green asterisks indicate magnocellular OXT neurons and green arrows indicate parvocellular OXT neurons. Yellow asterisks and white arrows denote magnocellular and parvocellular OXT neurons, respectively, that are colocalized with cholinergic neuron markers. Scale bars: 50 μm. Images are representative of adult fish (*n*=4; one fish per experiment).

Next, FISH experiments were carried out using probes for choline acetyltransferase genes (*chata* and *chatb*) to assess the cholinergic characteristics of OXT neurons in the hypothalamic POA. No *chata* signals (red) were observed colocalized with GFP-labeled OXT neurons (green) in any of the examined regions (PPa, PPp and TPp) (Fig. 3Ai–Di). In contrast, *chatb* signals (red) were colocalized with GFP-labeled OXT neurons (green) in the PPa region, confirmed in orthogonal *x*–*z* and *y*–*z* projections (Fig. 3Aii). Both predominant magnocellular (yellow asterisks) and parvocellular OXT neurons (white arrows) in the PPp and TPp regions also exhibited *chatb* expression (Fig. 3Bii–Dii). These findings were further substantiated by *z*-stack sectional analyses (Fig. 3Bii–Dii), which confirmed intracellular colocalization across all examined regions. These analyses were performed in adult fish (*chata*: *n*=4 fish; *chatb*: *n*=4 fish).

**Fig. 4.**
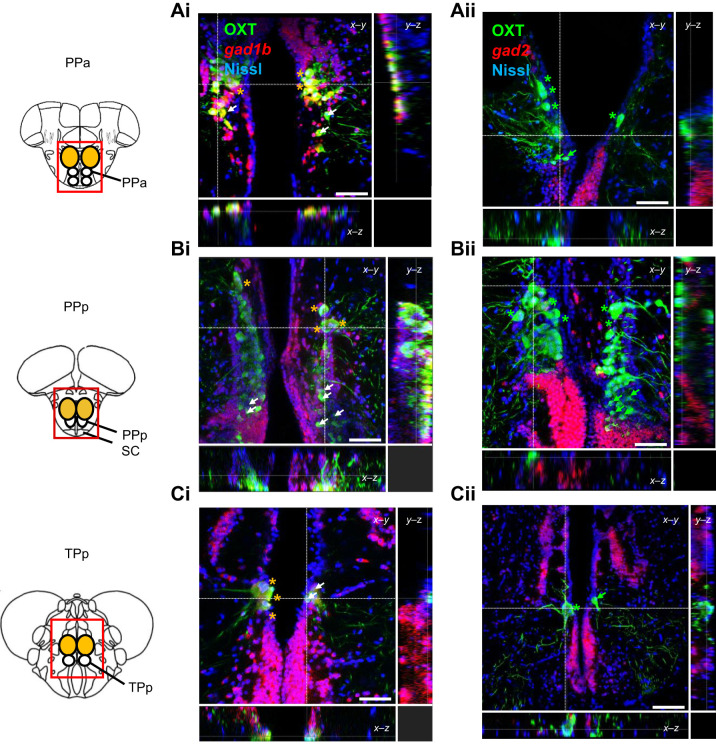
**OXT neurons co-express GABA across the preoptic and the periventricular nucleus of the posterior tuberculum.** Representative confocal *z*-stack images (with orthogonal projections) of the (Ai,ii) PPa, (Bi,ii) PPp and (Ci,ii,Di,ii) TPp showing OXT neurons (green) co-labeled with the GABAergic neuron marker *gad1b* (red; Ai,Bi,Ci,Di) but not *gad2* (red; Aii,Bii,Cii,Dii), with Nissl counterstain (blue). Green asterisks indicate magnocellular OXT neurons and green arrows indicate parvocellular OXT neurons. Yellow asterisks and white arrows denote magnocellular and parvocellular OXT neurons, respectively, that are colocalized with GABAergic neuron markers. Scale bars: 50 μm. Images are representative of adult fish (*n*=4; one fish per experiment).

We subsequently examined gamma-aminobutyric acid (GABA) neurotransmitter co-expression by targeting glutamic acid decarboxylase genes *gad1b* and *gad2*, which encode the rate-limiting enzymes for GABA production. In the PPa region, all GFP-labeled OXT neurons (green; Fig. 4Ai) displayed strong colocalization with *gad1b* signals (red; Fig. 4Ai), a finding confirmed through examination of orthogonal *x*–*z* and *y*–*z* sections. The morphology of these neurons, marked by white arrows, identified them predominantly as parvocellular types. In the PPp area, both the magnocellular OXT population (highlighted by yellow asterisks) and the parvocellular group exhibited similar *gad1b* colocalization (Fig. 4Bi), as verified with orthogonal projections. In the TPp, this pattern persisted, with *gad1b* detected in both magnocellular and parvocellular OXT neurons (Fig. 4Ci). In contrast to *gad1b*, no colocalization of *gad2* signals (red) with GFP-labeled OXT neurons (green) was detected in the PPa, a further result corroborated by orthogonal *x*–*z* and *y*–*z* projections (Fig. 4Aii). This lack of gad2 expression persisted in both magnocellular and parvocellular OXT neuronal populations within both PPp and TPp regions (Fig. 4Bii,Cii). These analyses were performed in adult fish (*gad1b*: *n*=4 fish; *gad2*: *n*=4 fish).

**Fig. 5.**
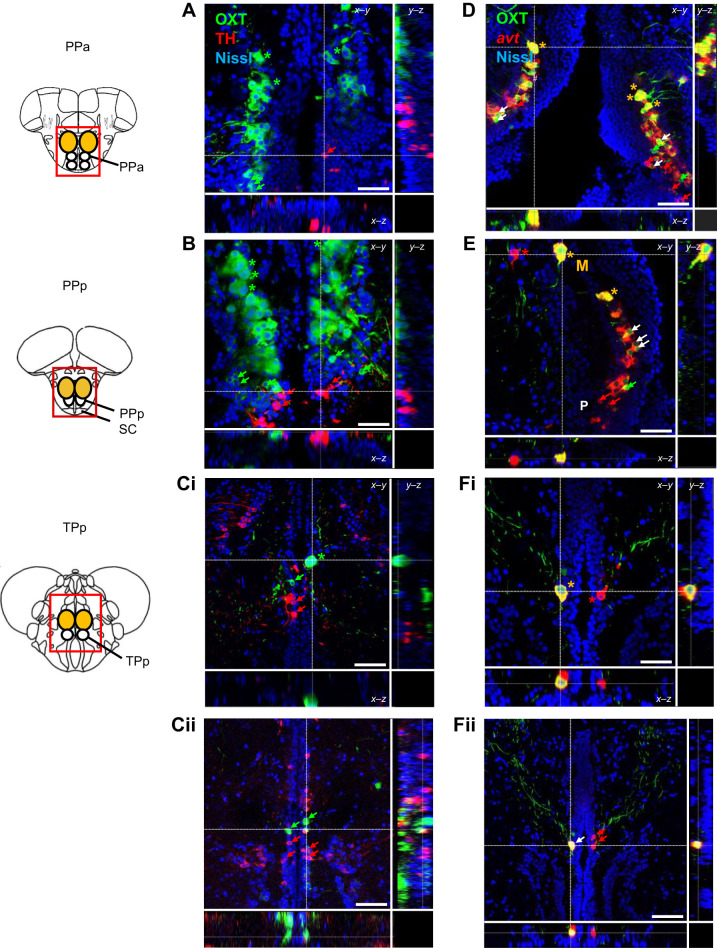
**OXT neurons lack dopamine synthesis markers but partially co-express vasotocin (AVT) across the preoptic and the periventricular nucleus of the posterior tuberculum.** Representative confocal *z*-stack images (with orthogonal projections) of the (A,D) PPa, (B,E) PPp and (Ci,ii,Fi,ii) TPp showing OXT neurons (green) do not colocalize with the dopamine synthesis marker tyrosine hydroxylase (TH) (red; A,B,Ci,ii) but partially colocalize with *avt* (red; D,E,Fi,ii), with Nissl counterstain (blue). Green asterisks indicate magnocellular OXT neurons and green arrows indicate parvocellular OXT neurons. Yellow asterisks and white arrows denote magnocellular and parvocellular OXT neurons, respectively, that are colocalized with AVT. Scale bars: 50 μm. Images in A–Ci,ii and D–Fi,ii are exemplary images from adult fish (=2 and 4, respectively; one fish per experiment).

We further examined whether OXT neurons exhibit characteristics of dopaminergic neurons. Double IHC analysis revealed that TH-immunoreactive signals (red) did not colocalize with GFP-labeled OXT neurons (green) in the PPa region (Fig. 5A), a finding further confirmed by orthogonal *x*–*z* and *y*–*z* projections. The number of dopaminergic (TH-positive) neurons was substantially lower than that of OXT neurons in the PPa. Neither magnocellular OXT neurons in the PPp nor parvocellular OXT neurons in the PPp showed TH signal colocalization (Fig. 5B). In addition, TH-positive neurons were primarily detected in the suprachiasmatic (SC) nucleus, and very few were present in the PPp region (Fig. 5B). In the TPp, orthogonal projections further demonstrated that neither subtype of OXT neurons exhibited colocalization with TH-positive cells (Fig 5Ci,ii). These analyses were performed in adult fish (TH: *n*=2 fish).

It is known that OXT and AVP are evolutionarily related nonapeptides that arose from a common ancestral vasotocin peptide through gene duplication, and considering their documented functional interactions in vertebrate social and neuroendocrine systems, we investigated whether zebrafish OXT neurons co-express AVT. We found that *avt* transcripts (red) were partially colocalized with OXT neurons (green) in the PPa, as confirmed by orthogonal *x*–*z* and *y*–*z* sections (Fig. 5E). Approximately 60% of *avt* signals overlapped with parvocellular OXT neurons (arrows) in this region (Fig. 5D). In the PPp, the majority of magnocellular OXT neurons (∼60%) displayed *avt* colocalization, whereas ∼25% parvocellular OXT neurons were *avt*-positive cells (Fig. 5E). These patterns of colocalization in the PPp were also confirmed by orthogonal projection analysis (Fig. 5E). In the TPp, both magnocellular and parvocellular OXT neurons were co-expressed with *avt* signals in the TPp region, as confirmed by orthogonal projections (Fig. 5Fi,ii). In addition, we also detected a few *avt* signals (red asterisks and red arrows) that were not co-expressed with OXT neurons in the TPp. These analyses were performed in adult fish (*avt*: *n*=4 fish).

**Fig. 6.**
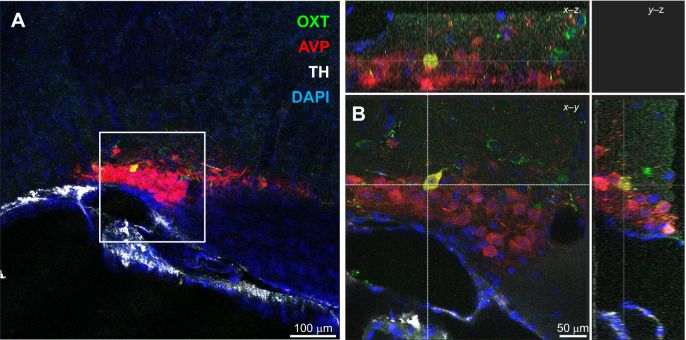
**OXT and AVP neurons are primarily segregated into distinct neuronal populations in the mouse SON, with rare colocalization.** (A) Representative confocal images of the mouse supraoptic nucleus (SON) showing the spatial distribution of OXT neurons (green), AVP neurons (red), tyrosine hydroxylase (TH, dopamine synthesis marker, white) and DAPI nuclear counterstain (blue). (B) Representative high-magnification confocal *z*-stack image (with orthogonal projections) of the SON showing a rare OXT neuron (green) that colocalizes with AVP (red). Images are representative of adult mice (*n*=3; one mouse per experiment).

We also examined OXT and AVP neurons in the mouse SON and PVN (Fig. 6; Fig. S1). Within the SON, the majority of OXT neurons (green; Fig. 6A) did not show colocalization with AVP signals (red; Fig. 6A), although a small subset exhibited co-expression (Fig. 6B). In the PVN, most OXT neurons lacked AVP signal (Fig. S1A), and only rare instances of colocalization were detected (Fig. S1B,C). These analyses were performed in adult mice (*n*=3 mice).

### Differential p-S6 labeling in parvocellular and magnocellular OXT neurons associated with mating behavior

**Fig. 7.**
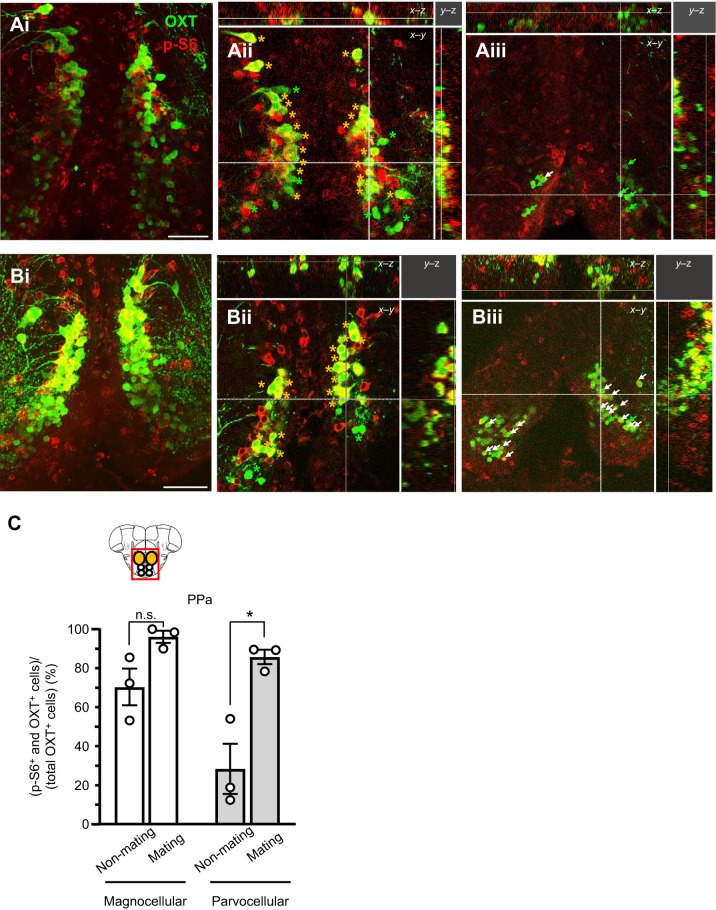
**Selective activation of OXT neuron populations in the PPa during mating behavior.** Representative confocal images of OXT neuron populations in the PPa from non-mating (Ai,ii,iii) and mating (Bi,ii,iii) zebrafish showing OXT neurons (green) and phospho-S6 immunoreactivity (p-S6, red) as a marker of translational activity and neuronal activation. In Aii and Aiii, green asterisks indicate magnocellular OXT neurons and green arrows indicate parvocellular OXT neurons. Yellow asterisks and white arrows indicate magnocellular and parvocellular OXT neurons, respectively, that colocalize with p-S6 signals. Scale bar: 50 μm. High-magnification confocal *z*-stack panels Aii and Bii depict magnocellular-enriched regions of the PPa, whereas Aiii and Biii depict parvocellular-enriched regions, allowing visualization of p-S6 activation patterns within each OXT neuron subtype. (C) Quantitative analysis of p-S6 immunoreactivity in magnocellular and parvocellular OXT neurons in the PPa during non-mating versus mating conditions. Data are presented as means±s.e.m. (two-tailed unpaired Student's *t*-test; *P<0.05; n.s., not significant). All data and images were obtained from adult fish (*n*=3; one fish per experiment).

**Fig. 8.**
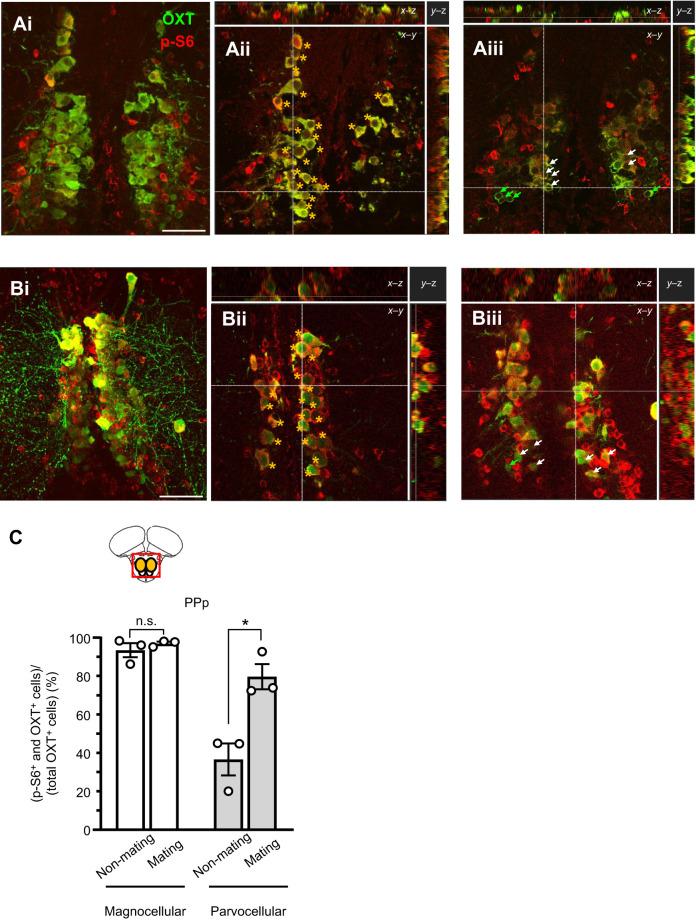
**Selective activation of OXT neuron populations in the PPp during mating behavior.** Representative confocal images of OXT neuron populations in the PPp from non-mating (Ai,ii,iii) and mating (Bi,ii,iii) zebrafish showing OXT neurons (green) and phospho-S6 immunoreactivity (p-S6, red) as a marker of translational activity and neuronal activation. In Aiii, green arrows indicate parvocellular OXT neurons. Yellow asterisks and white arrows indicate magnocellular and parvocellular OXT neurons, respectively, that colocalize with p-S6 signals. Scale bar: 50 μm. High-magnification confocal *z*-stack panels Aii and Bii depict magnocellular-enriched regions of the PPp, whereas Aiii and Biii depict parvocellular-enriched regions, allowing visualization of p-S6 activation patterns within each OXT neuron subtype. (C) Quantitative analysis of p-S6 immunoreactivity in magnocellular and parvocellular OXT neurons in the PPp during non-mating versus mating conditions. Data are presented as means±s.e.m. (two-tailed unpaired Student's *t*-test; *P<0.05; n.s., not significant). All data and images were obtained from adult fish (*n*=3; one fish per experiment).

**Fig. 9.**
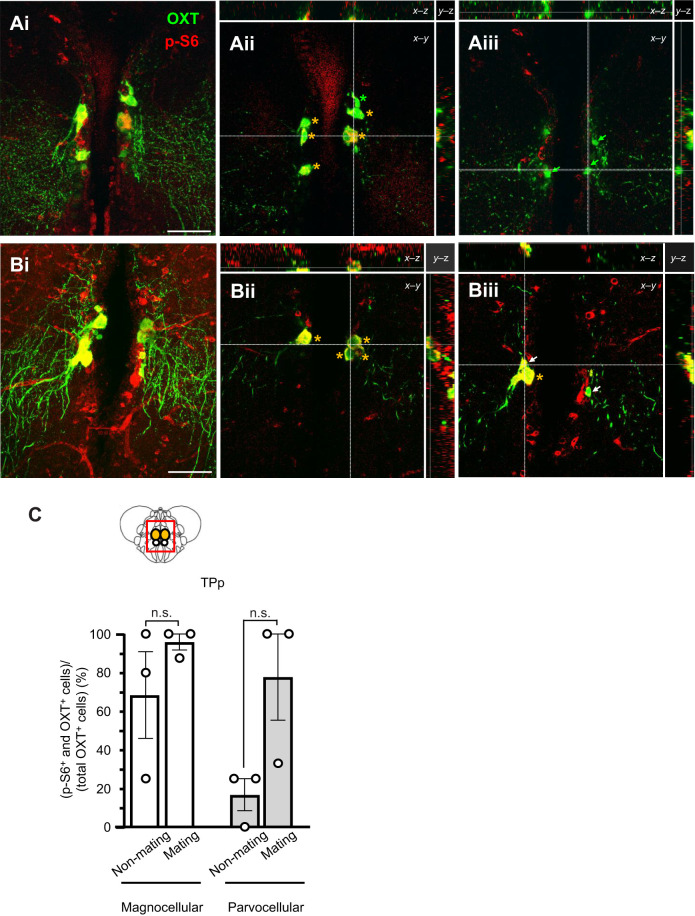
**Selective activation of OXT neuron populations in the TPp during mating behavior.** Representative confocal images of OXT neuron populations in the TPp from non-mating (Ai,ii,iii) and mating (Bi,ii,iii) zebrafish showing OXT neurons (green) and phospho-S6 immunoreactivity (p-S6, red) as a marker of translational activity and neuronal activation. In Aii and Aiii, green asterisks indicate magnocellular OXT neurons and green arrows indicate parvocellular OXT neurons. Yellow asterisks and white arrows indicate magnocellular and parvocellular OXT neurons, respectively, that colocalize with p-S6 signals. Scale bar: 50 μm. High-magnification confocal *z*-stack panels Aii and Bii depict magnocellular-enriched regions of the TPp, whereas Aiii and Biii depict parvocellular-enriched regions, allowing visualization of p-S6 activation patterns within each OXT neuron subtype. (C) Quantitative analysis of p-S6 immunoreactivity in magnocellular and parvocellular OXT neurons in the TPp during non-mating versus mating conditions. Data are presented as means±s.e.m. (two-tailed unpaired Student's *t*-test; n.s., not significant). All data and images were obtained from adult fish (*n*=3; one fish per experiment).

To assess OXT neuron activity during mating, we used Tg(*oxt*:EGFP) zebrafish to visualize OXT neurons (green fluorescence). Confocal microscopy was performed to capture *z*-stack images of OXT neurons across the PPa, PPp and TPp. p-S6 immunoreactivity was used as a marker of neuronal activity during mating behavior, as in previous studies that used p-S6 to measure socially induced neural activation in adult zebrafish (Kelly, 2019; Nunes et al., 2021). After mating, when brains were collected 50 min after the end of the mating trial, the proportion of p-S6-labeled OXT parvocellular neurons in the PPa was significantly increased compared with non-mated controls, which were taken directly from their home tanks and did not exhibit mating behavior under these housing conditions (40 fish per 10 l; spontaneous spawning is rare at this density) (non-mating: 28.37±12.95%; mating: 84.01±4.53%; *n*=3, *P*=0.012), corresponding to an approximately 2.96-fold increase (Fig. 7C). In the PPp, the proportion of p-S6-labeled OXT parvocellular neurons also increased by approximately 2.18-fold under the same comparison (non-mating: 36.63±8.31%; mating: 79.67±6.52%; *n*=3, *P*=0.015) (Fig. 8C). Confocal *z*-stack analysis with orthogonal *x*–*z* and *y*–*z* projections confirmed intracellular colocalization of p-S6 and GFP signals in parvocellular neurons (Figs 7Aiii,Biii, 8Aiii,Biii). In contrast, magnocellular OXT neurons in the PPa and PPp exhibited no significant change in p-S6 immunoreactivity between non-mating and mating groups. In the PPa, p-S6 labeled OXT magnocellular neurons showed comparable levels in both groups (non-mating: 70.38±9.37%; mating: 99.24±0.62%; *n*=3, *P*=0.059) (Fig. 7C). Similarly, in the PPp, p-S6 labeled OXT magnocellular neurons displayed no significant difference between mating and non-mating groups (non-mating: 93.51±3.71%; mating: 96.97±0.88%; *n*=3, *P*=0.41) (Fig. 8C). Orthogonal projections confirmed robust p-S6 labeling in magnocellular OXT neurons (Figs 7Aii,Bii, 8Aii,Bii). In the TPp, neither magnocellular (non-mating: 68.83±22.42%; mating: 95.83±4.17%; *n*=3, *P*=0.294) nor parvocellular OXT neurons (non-mating: 16.67±8.33%; mating: 77.67±22.33%; *n*=3, *P*=0.062) showed a similar pattern in p-S6 immunoreactivity during mating behavior (Fig. 9C). Detailed absolute counts for each brain region and OXT neuron subtype are reported in Table S1.

## DISCUSSION

OXT is a phylogenetically conserved neuropeptide mediating social, reproductive and stress-related behaviors across vertebrates (Grinevich et al., 2016; Insel, 2010; Knobloch and Grinevich, 2014). In mammals, magnocellular and parvocellular OXT neuron populations in the SON and PVN exhibit distinct projections and neuroendocrine roles (Althammer and Grinevich, 2017). Although magnocellular and parvocellular OXT neurons have been identified in teleost fish, their precise anatomical distribution, neurochemical diversity and differential recruitment during specific social behaviors in the adult brain remain incompletely characterized (Herget et al., 2014; Mennigen et al., 2022; Reddon et al., 2017; Saito et al., 2004; Wee et al., 2019; Wircer et al., 2017). The present study addressed this gap by providing the first systematic characterization and quantification of OXT neuron morphotypes and their multi-neurotransmitter co-expression patterns in the adult zebrafish brain. We demonstrate that despite possessing substantially fewer OXT neurons than mammals, zebrafish achieve behavioral complexity through OXT neurons with distinct morphologies and co-expressed neurotransmitters. These findings suggest that limited OXT neuron populations are functionally expanded through co-release of multiple neuroactive substances, a cellular strategy that may be shared across vertebrate lineages with divergent neuronal resources.

In the present study, we identified a rostrocaudal gradient in OXT neuron density, with the largest population in the PPa, followed by the PPp, and substantially fewer neurons in the TPp (Fig. 1B). This distribution is consistent with observations in other teleost species, including European plaice (*Pleuronectes platessa*), green molly (*Poecilia latipinna*), plainfin midshipman (*Porichthys notatus*), gulf toadfish (*Opsanus beta*), rainbow trout (*Oncorhynchus mykiss*) and white sea bream (*Diplodus sargus*), which all exhibit predominant OXT neurons in the PPa with progressively sparser populations in the PPp (Batten et al., 1990; Duarte et al., 2001; Goodson et al., 2003; Saito et al., 2004). Furthermore, our quantitative analysis provides the first systematic characterization of OXT neuron subtype composition across the POA and TPp, revealing the regional differences. Magnocellular neurons comprised 22.31% of the OXT population in the PPa, 44.51% in the PPp and 59.26% in the TPp (Fig. 1C–E), revealing a rostrocaudal gradient in magnocellular proportion. However, the TPp contained relatively few OXT neurons overall (*n*<20 per fish), indicating that the gradient reflects proportional enrichment rather than absolute neuronal abundance. In contrast to mammalian PVN and SON, zebrafish POA exhibits a distinctive morphotype distribution. Within the POA, parvocellular neurons dominated regions (PPa: 78%; PPp: 55%; Fig. 1C,D), a distribution pattern opposite to mammalian PVN (approximately 25–30% parvocellular) and SON (approximately 0% parvocellular) (Lewis et al., 2020; Sabatier et al., 2013). Although the TPp magnocellular proportion (59%; Fig. 1E) is similar to mammalian PVN (∼70%), TPp represents a teleost-specific nucleus that lacks a clear mammalian homolog based on developmental origin, anatomical position and projection patterns. Given the limited number of OXT neurons in TPp, the precise function of this caudal population remains to be determined.

We further observed a dorsoventral segregation of OXT neurons within each preoptic nucleus. Magnocellular neurons were enriched in the dorsal subdivisions of the PPa and PPp, whereas parvocellular neurons were concentrated in ventral regions, with an intermediate zone exhibiting mixed populations of both subtypes (Fig. 1C,D). This dorsoventral organization is consistent with observations in other teleost species, including rainbow trout (*Oncorhynchus mykiss*) (Saito et al., 2004), white sea bream (*Diplodus sargus*) (Duarte et al., 2001) and plainfin midshipman (*Porichthys notatus*) (Goodson et al., 2003), suggesting a conserved teleost arrangement. Mammals evolved a different spatial pattern through nuclear segregation. The SON contains exclusively magnocellular neurons (Sofroniew, 1980). The PVN, by contrast, contains both cell types but displays a reversed spatial organization: magnocellular neurons located in lateral and posterolateral regions and parvocellular neurons in medial and dorsal regions (Sawchenko and Swanson, 1982; Swanson and Kuypers, 1980). This spatial and functional segregation enables independent regulation of systemic and central OXT functions (Sawchenko and Swanson, 1982; Swanson and Kuypers, 1980). The limited OXT neuron population in zebrafish may represent a more ancestral state compared with mammals. In contrast, mammals have undergone significant anatomical and functional elaboration of the OXT system, with distinct neuronal populations specialized for distinct physiological states. Although glutamatergic neuron markers have been identified in the zebrafish POA, colocalization with OXT neurons has not been demonstrated (Hiraki-Kajiyama et al., 2024). Our confocal analysis with orthogonal projections directly demonstrated colocalization of *slc17a6a* and *slc17a6b* in all OXT neuron subtypes (Fig. 2). This glutamatergic characteristic in teleost OXT neurons parallels findings in mammals. Magnocellular OXT neurons in the SON and PVN co-express VGLUT2 (Hrabovszky and Liposits, 2008; Ponzio et al., 2006), indicating that this glutamatergic co-expression with OXT neurons is conserved in vertebrates. Although the functional significance remains unexplored, evidence from analogous systems suggests this co-transmission could enhance circuit plasticity (Nusbaum et al., 2017). Direct functional evidence in both mammals and teleosts is currently lacking.

In addition to widespread glutamatergic co-expression (*slc17a6a/b*), we also identified cholinergic (*chatb*) and GABAergic (*gad1b*) neuron markers in both magnocellular and parvocellular OXT neurons (Figs 3, 4). These results show that OXT neurons express multiple neurotransmitters in addition to oxytocin, representing a previously undescribed neurochemical characteristic. This combination of neurotransmitters may enable complex neuromodulation. In the mammalian forebrain, for example, cholinergic neurons that co-release GABA produce state-dependent control over cortical excitability (Saunders et al., 2015), suggesting a potential model for how OXT neurons could coordinate multiple signaling pathways.

Despite extensive studies of OXT characteristics across vertebrate species, direct evidence for colocalization of oxytocin with dopaminergic or serotonergic neuron markers remains limited. In humans, one study reported colocalization of TH with OXT in the PVN and SON (Panayotacopoulou et al., 1994), though this finding has not been replicated in other mammalian species. Recent comprehensive single-neuron profiling in mice found no evidence of dopaminergic or serotonergic neuron marker co-expression within OXT subpopulations (Li et al., 2024). In the present study, we extend these observations to the mouse, where double-immunofluorescence labeling of the PVN and SON revealed that OXT neurons predominantly lack dopaminergic neuron markers (Fig. 6). In teleosts, whether OXT neurons co-express TH or serotonergic markers remained unexamined. Our results provide direct evidence that OXT neurons lack both TH (Fig. 5) and serotonergic (data not shown) synthesis markers, despite co-expressing multiple other classical neurotransmitters, including glutamate, acetylcholine and GABA (Figs 2–5).

In early jawed vertebrates, OXT- and AVP-like peptides are thought to have been co-expressed in the same neurons. Following gene duplication, these neurons are proposed to have diverged into separate OXT- or AVP-expressing cell types through a duplication–degeneration–complementation process (Gilligan et al., 2003). Within this framework, AVP likely retained its ancestral role in osmotic and cardiovascular homeostasis, whereas OXT was co-opted to support allostatic control of stress, social behavior and adaptive energetics, leading to broader functional diversification across vertebrate taxa (Leng and Russell, 2019; Takayanagi and Onaka, 2022). In zebrafish, OXT and AVT systems influence overlapping physiological and behavioral processes, including fluid balance and defensive responses (Wee et al., 2019). Dual-peptide neurons may therefore provide a substrate for coordinating multiple internal state variables. In the present study, we found that a subset of zebrafish OXT neurons in the PPa, PPp and TPp co-express *avt* (Fig. 5D,E,Fi,ii).

By contrast, mammalian PVN and SON showed minimal OXT–AVP colocalization, with overlap restricted to only a small minority of cells (Fig. 6; Fig. S1; Mezey and Kiss, 1991; Soumier et al., 2021). The degree of OXT–AVT segregation varies substantially across vertebrates. These findings raise an important evolutionary question about whether the partial OXT–AVT co-expression we observe in zebrafish reflects a retained ancestral organization or a lineage-specific adaptation. At present, this question cannot be resolved from a single species alone. Broader comparative analyses across additional teleost species will be required to determine whether this pattern is conserved, how it relates to preoptic neuron size classes and projection patterns, and whether OXT–AVT double-positive neurons represent a shared ancestral feature or a zebrafish-specific specialization.

We examined whether OXT neuron subpopulations show differential activity during mating. Both the PPa and PPp parvocellular OXT neurons, but not magnocellular neurons, exhibited higher p-S6 labeling in mating fish than in non-mating controls (Figs 7, 8). These findings imply that teleost parvocellular and magnocellular OXT neurons might be differentially recruited during mating-related behavior. Nevertheless, because the control fish were socially housed and were not completely devoid of social stimuli, we cannot rule out contributions from non-reproductive social cues, such as visual or olfactory input, or from stress associated with the testing context to the observed p-S6 response. In mammals, activation of OXT neuron subpopulation during mating is more complex. Both parvocellular and magnocellular OXT neurons in the PVN can be activated following copulation in rats (Veening et al., 2015; Witt and Insel, 1994). However, in female rats, only parvocellular OXT neurons showed heightened responsiveness to intromissive mating stimulation, whereas magnocellular OXT neurons remained inactive (Polston et al., 1998). These parvocellular neurons project to brainstem and spinal regions to coordinate reproductive physiologies and behaviors (Oti et al., 2020). This pattern may reflect functional specialization in teleosts relative to mammals, possibly shaped by evolutionary differences in reproductive strategies. These findings are consistent with a model in which magnocellular neurons may function on a slower endocrine timescale to support systemic readiness, whereas parvocellular neurons may provide more rapid, behavior-dependent modulation of central reproductive circuits (Brown et al., 2013; Mennigen et al., 2022; Veening et al., 2015).

In contrast to the PPa and PPp, OXT neurons in the TPp region showed no significant p-S6 induction during mating in either magnocellular or parvocellular neurons (Fig. 9). The TPp occupies a more caudal and medial position within the basal hypothalamus. It belongs to the posterior tuberculum, which differs from the preoptic regions containing the PPa and PPp (Schredelseker and Driever, 2020). Unlike the PPa and PPp, the TPp lacks a clear dorsoventral organization. This anatomical distinction supports the idea that the TPp OXT neurons may serve functions different from those of preoptic parvocellular neurons, potentially contributing to longer-timescale neuroendocrine integration, such as seasonal reproductive preparation, osmotic homeostasis or stress-related hypothalamic regulation, rather than to the acute coordination of mating behavior. A previous study using calcium imaging and z-ERK activity mapping in zebrafish has shown that hypothalamic OXT neurons display rapid and diverse activity patterns during social or aversive situations (Wee et al., 2019), whereas our p-S6 analysis in adults instead captured which anatomically defined OXT subpopulations are more strongly engaged over time during mating behavior.

In the present study, we measured p-S6 only in OXT-labeled neurons and did not include neurotransmitter markers, which is an important limitation. As a result, we can compare how parvocellular and magnocellular OXT neurons are recruited during mating, but we cannot yet tell which neurotransmitter-defined OXT subtypes are preferentially involved. Additional studies using p-S6 together with neurotransmitter markers, and possibly electrophysiology with pharmacological manipulations, will be required to directly link neurotransmitter co-expression to mating-related activity.

### Conclusions

Zebrafish may achieve sophisticated reproductive control through state-dependent circuit engagement and multi-neurotransmitter co-release, despite maintaining a smaller OXT neuronal population than mammals. Parvocellular OXT neurons in the PPa and PPp may be involved in mating behavior, whereas magnocellular neurons show little change in p-S6 labeling under the same conditions. Individual OXT neurons co-express glutamate, GABA and acetylcholine, conspicuously lacking dopaminergic and serotonergic markers. This selective co-expression may enable parvocellular neurons to exert multi-dimensional control. Unlike mammals that elaborate the OXT system through population expansion, zebrafish achieve equivalent complexity through neurotransmitter versatility in individual neurons. The TPp OXT neurons showed no mating-dependent activation, suggesting a distinct role in slower physiological processes such as pre-mating motivation or post-mating recovery. Future studies should map parvocellular projections and characterize TPp engagement during diverse social behaviors.

## Supplementary Material

10.1242/jexbio.252228_sup1 Supplementary information
