# Correction: Multi-transmitter characteristics and functional specialization of oxytocin neuron subpopulations in zebrafish

**DOI:** 10.1242/jeb.253271

**Published:** 2026-08-14

**Authors:** Sian-Tai Liu, Bo-Kai Liao, Meng-Shin Shiao, Yen-Lin Chen, Yu-Fan Huang, Ming-Yi Chou

There was an error in *J. Exp. Biol.* (2026) **229**, jeb252228 (doi:10.1242/jeb.252228).

The surname of author Meng-Shin Shiao was spelt incorrectly (Meng-Shin Shaio). The correct name appears above and both the online full-text and PDF versions of the article have been updated.

The authors apologise for this error and any inconvenience it may have caused.

