# Supplementary material for: Multi-transmitter characteristics and functional specialization of oxytocin neuron subpopulations in zebrafish
Source: J Exp Biol. 2026 May 13;229(9):jeb252228. doi: 10.1242/jeb.252228 (PMC13245913; doi:10.1242/jeb.252228)
Supplement: Supplementary information [file jexbio-229-252228-s1.pdf]

Supplementary Materials and Methods

Vibratome sectioning parameters

Following fixation, adult zebrafish brains were rinsed thoroughly in PBS and embedded in 2% agarose prepared in PBS. The agarose solution was heated to 65–70 °C until fully dissolved and cooled to approximately 40 °C prior to embedding. Individual brains were positioned in plastic molds (15 × 10 × 10 mm) with the rostro-caudal axis aligned parallel to the mold base and allowed to solidify at room temperature.

Serial coronal sections were generated using a vibrating microtome (Leica VT1200S, Leica Microsystems) equipped with Feather Double Edge stainless steel blades (Feather Safety Razor Co., Ltd.). Section thickness was set to 80 µm for specimens processed for fluorescence in situ hybridization (FISH) and 100 µm for specimens processed for immunohistochemistry (IHC). Vibratome settings for zebrafish tissue were optimized as follows: oscillation frequency, 50 Hz; oscillation amplitude, 0.75 mm; and blade advance speed, 0.4 mm/s. Sections were collected into PBS and stored at 25 °C for up to 7 days prior to further processing.

For mouse brains, coronal sections (80 µm) were obtained using a vibratome (Campden Instruments, UK) following overnight post-fixation in 4% PFA at 4 °C.

Fluorescence *in situ* hybridization (FISH) protocol and RNA probe synthesis

Total RNA was extracted from adult zebrafish brains using TRIzol reagent (Invitrogen) according to the manufacturer's instructions. First-strand cDNA was synthesized using SuperScript IV Reverse Transcriptase (Thermo Fisher, 18090050). DIG-labelled antisense RNA probes were generated from cDNA templates using T7 RNA polymerase (Roche, RPOL T7-RO) and the Roche DIG RNA Labeling Kit (11175025), then purified using the RNeasy Mini Kit (Qiagen, 74106) and quantified by NanoDrop spectrophotometry.

Probes targeted the following transcript regions (GenBank accession numbers in parentheses): *slc17a6a*, nucleotides 138-967 (NM_001009982.1); *slc17a6b*, nucleotides 466-1050 (NM_001128821.1); *chata*, nucleotides 714-1374 (XM_021480489.1); *chatb*, nucleotides 374-1187 (NM_001291882); *gad1b*, nucleotides 583-1330 (NM_194419.1); *gad2*, nucleotides 4026-4837 (NM_001017708.2); and *avt*, nucleotides 5-454 (NM_178293.2).

Tissue pretreatment and hybridization

Fixed vibratome sections were subjected to a graded methanol dehydration series at room temperature: 75% PBS/25% methanol (v/v) for 5 min, 50% PBS/50% methanol for 5 min, 25% PBS/75% methanol for 5 min, and 100% methanol for 5 min. This

Journal of Experimental Biology • Supplementary information

dehydration cycle in 100% methanol was repeated once more, and samples were stored at −20 °C for at least 2 h to ensure complete solvent exchange. Endogenous peroxidase activity was quenched by incubating sections in 3% hydrogen peroxide (H_2O_2) in 100% methanol for 1 h at room temperature.

Sections were then rehydrated through a reverse methanol gradient into PBS with 0.1% Triton X-100 (PBSTr): 75% methanol/25% PBSTr, 50% methanol/50% PBSTr, and 25% methanol/75% PBSTr (5 min each), followed by two washes in 100% PBSTr. To enhance probe penetration, sections were incubated with proteinase K (10 µg/mL in PBSTr; Sigma, 124568) for 20 min at room temperature. Digestion was terminated by washing in PBSTr and re-fixation in 4% PFA at 4 °C for 20 min.

Sections were equilibrated in hybridization buffer (HyB) composed of 60% formamide, 25 % 20× SSC, 0.1% Tween-20, 5% dextran sulfate, 2.5 mg/mL torula yeast tRNA, and 100 mg/mL heparin. Samples were pre-hybridized in HyB at 58 °C for at least 2 h. Fresh HyB containing DIG-labelled antisense riboprobes (approximately 100 ng total probe per sample) was then applied, and sections were incubated for 16 h at 58 °C to allow target-specific hybridization.

Post-hybridization washes and detection

Non-specifically bound probes were removed by a series of stringent washes at 58 °C using pre-warmed buffers. Sections were incubated in serial dilutions of HyB in 2× SSCT (2× SSC with 0.1% Tween-20), followed by three 15 min washes in 2× SSCT and three 15 min washes in 0.2× SSCT. Samples were then equilibrated to room temperature and washed three times in 10× PBSTr (5 min each).

To block non-specific antibody binding, sections were incubated in 1% blocking buffer (Roche, in 1× PBSTr) for 2 h at room temperature. DIG-labelled riboprobes were detected using Anti-digoxigenin-POD conjugate (Roche, Lot 74946000) diluted 1:400 in blocking buffer and incubated overnight at 4 °C. Signal amplification was achieved using a tyramide signal amplification (TSA) system: sections were incubated with CF Dye Tyramide 568 (Biotium, 92173) at 1:1,000 for 20 min at room temperature.

Following TSA, sections were washed six times in PBSTr (10 min each) at room temperature to remove unbound reagents. Nissl staining was performed using NeuroTrace 640/660 deep-red fluorescent stain (Thermo Fisher, N21483) diluted 1:1000 in PBSTr, applied for 30 min at room temperature in the dark to minimize photobleaching. Excess dye was removed by washing in PBSTr prior to confocal imaging.

Journal of Experimental Biology • Supplementary information

Immunohistochemistry (IHC) protocol

Zebrafish brain sections

For IHC on zebrafish brain sections, free-floating vibratome slices were transferred to 24-well plates and incubated in blocking buffer (Roche blocking reagent diluted in PBSTr) at room temperature for 2 h on a gentle orbital shaker. Primary antibodies were diluted in fresh blocking buffer at the following concentrations: chicken anti-GFP (Abcam, ab286193; 1:1000), mouse anti-tyrosine hydroxylase (TH; Sigma-Aldrich, MAB318; 1:500), and rabbit anti-phospho-S6 ribosomal protein (Cell Signaling Technology, 2211S; 1:500). Sections were incubated with 300 µL primary antibody solution per well at 4 °C overnight (12–16 h) with gentle agitation.

After primary incubation, sections were washed five times in PBSTr (10 min per wash) at room temperature on an orbital shaker to remove unbound antibodies. Secondary antibodies were diluted in 1× PBSTr: goat anti-chicken IgY Alexa Fluor 488 (Thermo Fisher, A-11039; 1:300) for GFP, goat anti-mouse IgG Alexa Fluor 568 (Thermo Fisher, A-11004; 1:300) for TH, and goat anti-rabbit IgG Alexa Fluor 568 (Thermo Fisher, A-11011; 1:300) for p-S6. Sections were incubated with 300 µL of secondary antibody solution per well for 2 h at room temperature in a light-protected humid chamber.

Following secondary incubation, sections were washed five times in PBSTr (10 min each) at room temperature. Nissl staining was performed using NeuroTrace 640/660 deep-red fluorescent stain (Thermo Fisher, N21483) diluted 1:1000 in PBSTr and applied for 30 min at room temperature in the dark, followed by PBSTr washes to remove excess dye. Sections were then mounted on glass slides with anti-fade mounting medium and coverslipped for confocal imaging.

Mouse brain sections

For mouse tissue, free-floating sections were incubated in blocking solution consisting of 10% horse serum in PBS with 0.3% Triton X-100 for 2 h at room temperature. Primary antibodies were diluted in the same blocking solution at optimized concentrations: guinea pig anti-oxytocin (Peninsula Laboratories, T-5021; 1:1,000), rabbit anti-vasopressin (Immunostar, AB_572219; 1:2,000), and chicken anti-tyrosine hydroxylase (Abcam, ab76442; 1:1,000). Sections were incubated with primary antibodies overnight at 4 °C on an orbital shaker.

After washing in PBS, sections were incubated for 2 h at room temperature with secondary antibodies diluted in PBS: goat anti-guinea pig Alexa Fluor 488 (Invitrogen; 1:500), goat anti-rabbit CF568A (Biotium; 1:500), and goat anti-chicken Alexa Fluor 633 (Invitrogen; 1:500). Sections were then washed, mounted on slides, and coverslipped using Vectashield HardSet mounting medium with DAPI (Vector Laboratories) to counterstain nuclei.

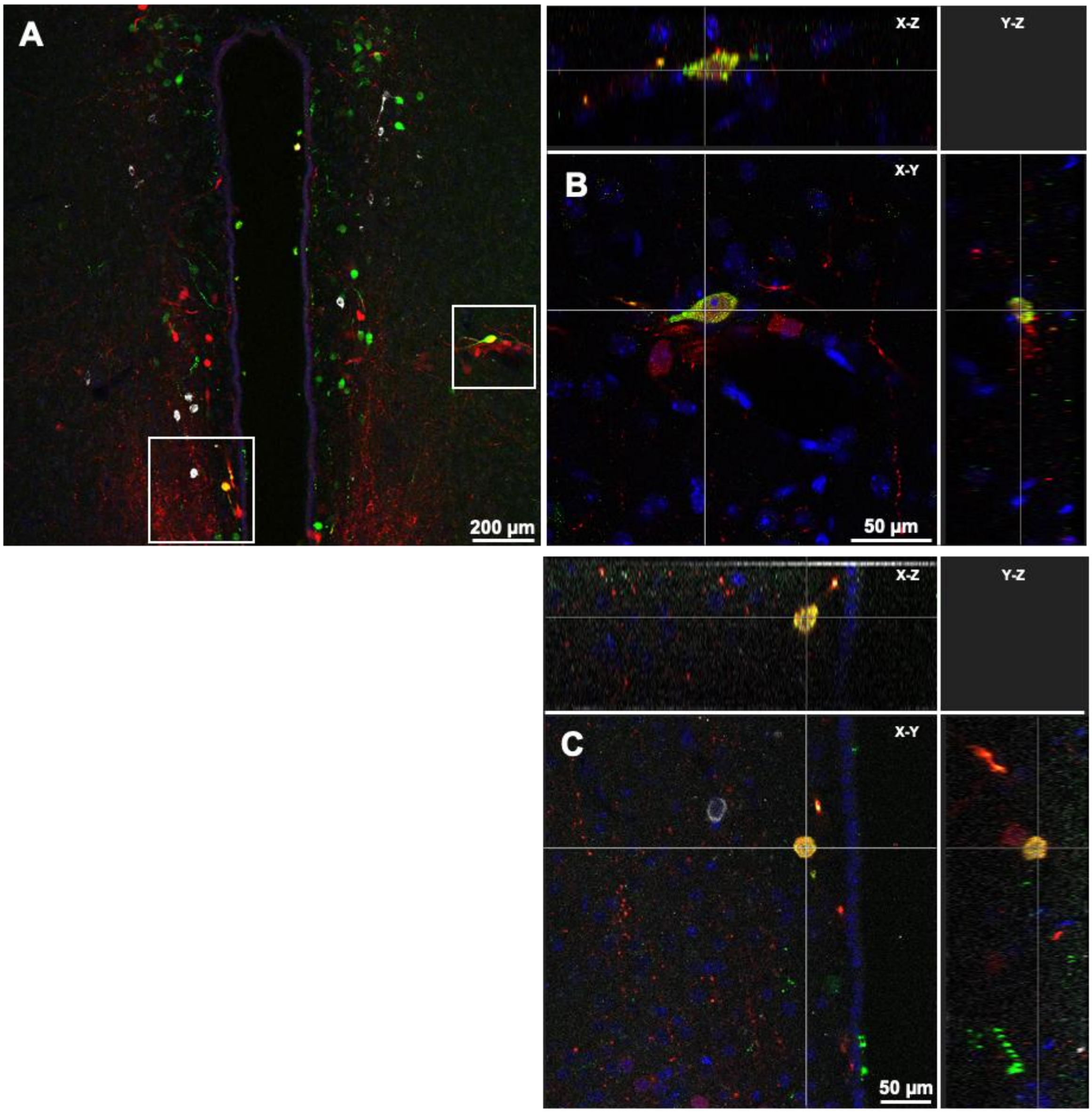

Fig. S1. OXT and AVP neurons are primarily segregated into distinct neuronal populations in the mouse PVN, with rare colocalization.

(A) Representative confocal images of the mouse paraventricular hypothalamus (PVN) showing the spatial distribution of OXT neurons (green), AVP neurons (red), tyrosine hydroxylase (TH, dopamine synthesis marker, white), and **DAPI** nuclear counterstain (blue). (B and C) Representative high-magnification confocal z-stack images (with orthogonal X-Z and Y-Z projections) of the PVN showing a rare OXT neuron (green) colocalizing with AVP (red). Images are representative of adult mice (n = 3).

Journal of Experimental Biology • Supplementary information

Table S1. Absolute numbers and percentages of p-S6 positive OXT neurons in mating and non-mating fish for each brain region and neuronal subtype.

Region	Subtype	Condition	Fish ID	OXT^+ cells	$pS6^+$ + OXT^+ cells	$pS6^+$ + OXT^+ cells (%)
PPa	Magnocellular	Non-mating	F1	29	21	72
PPa	Magnocellular	Non-mating	F2	96	59	86
PPa	Magnocellular	Non-mating	F3	62	33	53
PPa	Magnocellular	Mating	F1	23	23	100
PPa	Magnocellular	Mating	F2	66	65	98
PPa	Magnocellular	Mating	F3	91	82	90
PPa	Parvocellular	Non-mating	F1	170	21	12
PPa	Parvocellular	Non-mating	F2	96	18	19
PPa	Parvocellular	Non-mating	F3	35	19	54
PPa	Parvocellular	Mating	F1	195	153	78
PPa	Parvocellular	Mating	F2	67	60	90
PPa	Parvocellular	Mating	F3	56	50	89
PPp	Magnocellular	Non-mating	F1	50	48	96
PPp	Magnocellular	Non-mating	F2	29	25	86
PPp	Magnocellular	Non-mating	F3	56	50	98
PPp	Magnocellular	Mating	F1	50	49	98
PPp	Magnocellular	Mating	F2	84	80	95
PPp	Magnocellular	Mating	F3	44	43	97
PPp	Parvocellular	Non-mating	F1	91	41	45
PPp	Parvocellular	Non-	F2	50	10	20

		mating				
PPp	Parvocellular	Non-mating	F3	29	13	44
PPp	Parvocellular	Mating	F1	58	42	72
PPp	Parvocellular	Mating	F2	23	17	74
PPp	Parvocellular	Mating	F3	38	31	92
TPp	Magnocellular	Non-mating	F1	5	5	100
TPp	Magnocellular	Non-mating	F2	8	2	25
TPp	Magnocellular	Non-mating	F3	5	4	80
TPp	Magnocellular	Mating	F1	11	11	100
TPp	Magnocellular	Mating	F2	8	7	87
TPp	Magnocellular	Mating	F3	4	4	100
TPp	Parvocellular	Non-mating	F1	4	1	25
TPp	Parvocellular	Non-mating	F2	4	0	0
TPp	Parvocellular	Non-mating	F3	4	1	25
TPp	Parvocellular	Mating	F1	3	1	33
TPp	Parvocellular	Mating	F2	2	2	100
TPp	Parvocellular	Mating	F3	3	3	100
